# Factors affecting readmission of adolescent mental healthcare users to a psychiatric hospital

**DOI:** 10.4102/sajpsychiatry.v29i0.2110

**Published:** 2023-11-15

**Authors:** Stephanie A. Eichstadt, Shren Chetty, Thulisile G. Magagula, Xan Swart

**Affiliations:** 1 Department of Psychiatry, Faculty of Psychiatry, University of Pretoria, Pretoria, South Africa; 2 Private Practice, Pretoria, South Africa

**Keywords:** adolescent, mental health, readmission, risk factors, psychiatric hospital

## Abstract

**Background:**

Adolescent mental illness is increasing worldwide, leading to more admissions to psychiatric institutions. Many adolescents may require multiple readmissions, which is disruptive to their holistic well-being and costly for the healthcare sector. Identifying especially modifiable risk factors for readmission remains an important step in providing potential areas for improving patient care.

**Aim:**

This study investigated the risk factors associated with the readmission of adolescent mental healthcare users to a specialist psychiatric unit.

**Setting:**

The specialist adolescent unit at Weskoppies Psychiatric Hospital.

**Methods:**

In this retrospective study, the clinical files of 345 adolescents admitted between 2015 and 2019 were reviewed. The primary outcome variable was readmission, that is, whether a patient was readmitted to Weskoppies Hospital (*n* = 98) compared to those with no recorded readmission (*n* = 247).

**Results:**

Readmitted adolescents were significantly younger on first admission compared to the non-readmitted group (13.46 vs 14.26, *p* = 0.016). Bivariate analysis showed that the readmitted group had a much higher rate of non-adherence to treatment (38.1% vs 10.5%, *p* = < 0.001). Patients with a family history of mental illness had a significantly higher risk of readmission (52.2% vs 37.5%, *p* = 0.015).

**Conclusions:**

Adolescents were more likely to be readmitted if they had first admission at a younger age, a family history of mental illness or non-adherence to treatment.

**Contribution:**

Identifying especially modifiable risk factors for readmission of adolescents to improve patient care, particularly in the South African context where there is a paucity of research on this topic.

## Introduction

The number of adolescents suffering from mental health conditions is increasing worldwide.^1^ Although there is a concerted effort to manage adolescent patients in a community setting, some patients may need to be admitted to psychiatric hospitals for appropriate management. Many adolescents may require multiple readmissions,^2^ which is disruptive to their holistic well-being. Patients who are admitted to the hospital are absent from school, which limits their academic exposure and performance as well as reduces their peer connectedness and slows their social development.^3^ Adolescents who are admitted are often isolated from their families, which may cause significant emotional distress.^4^ Inpatient admission is also costly for the healthcare sector, with inpatient care currently representing 86% of the total mental healthcare expenditure in South Africa.^5,6^

Various risk factors for adolescent readmission have been identified with the aim of instituting preventive measures. Although some factors, such as patient demographics and clinical diagnoses, remain relatively static, modifiable factors provide a potential area for improving patient care and could reduce the overall risk for readmission.

Several studies have identified younger age at first admission as a risk factor for readmission.^7,8,9^ In contrast, a study done in Copenhagen found no such correlation.^10^ It has been suggested that male gender is protective against readmission,^3^ as is for achieving a higher level of education.^11^ However, few studies have investigated the association between readmission and different types of schooling, for example, mainstream schooling versus special schooling.

There is conflicting evidence on the association between readmission and the primary psychiatric diagnoses of patients. Some studies report similar risk for readmission across psychiatric diagnoses, while other studies suggest that certain diagnoses increase the risk of readmission.^8,12^ Readmission has been associated with the severity of psychiatric symptoms, including suicidality. Suicidal ideation or suicide attempts are known to indicate a high risk for readmission.^9,13,14,15^ Readmission is also associated with other clinical risk factors, including having a comorbid substance use disorder^16^ and a personal history of trauma, particularly of sexual assault.^8,17^

Family and socioeconomic factors have been demonstrated to play a role in readmission. Adolescents with a family history of mental illness seem to have a higher risk for readmission,^17,18^ while adolescents with a parent as their primary caregiver appear to have a lower risk.^18,19^ Unfortunately, in countries such as South Africa, there are many child-headed households or households with absent parents, which may increase the overall risk for psychiatric readmission.^20^ Few studies have investigated the influence of other living circumstances, such as living in conflictual home circumstances, on the risk of readmission. The living circumstances of adolescent psychiatric patients should be considered during treatment and post-discharge follow-up.

The risk for readmission can be reduced with timely outpatient follow-up,^21^ including the psychoeducation of patients and reiterating the importance of treatment adherence.^22^ A study by Bobier and Warwick highlights the importance of adherence; the authors found that 47% of readmitted patients in the study did not adhere to treatment, as compared to 11% of the non-readmitted group.^8^

The readmission of adolescents to psychiatric institutions is associated with varying modifiable and non-modifiable risk factors. However, there is a discordance in the literature concerning some factors, which limits the generalisability and comparability of the findings. There is a paucity of research conducted in a South African context, and little is known about which factors are associated with the readmission of adolescent mental healthcare users in an urban setting in South Africa. The authors investigate the factors associated with readmission of adolescent psychiatric inpatients and determine the proportion of adolescent patients admitted to the Weskoppies Psychiatric Hospital adolescent unit who were readmitted. Moreover, we identify potentially modifiable risk factors associated with being readmitted, which may have a pivotal impact on patient management and outcomes.

## Research methods and design

### Study design

This was a case-control study involving a retrospective review of patient clinical files; therefore, no personal patient consent was required.

### Study setting

The study was conducted at the specialist adolescent unit at Weskoppies Psychiatric Hospital, a tertiary-level psychiatric hospital in Tshwane, Gauteng, South Africa. Weskoppies Hospital’s adolescent unit receives referrals from both the private and public health sectors and provides multidisciplinary psychiatric services on an inpatient and outpatient basis. A large proportion of the patients have moderate-to-severe mental illness and are of lower- or middle-class socioeconomic status.

### Study population and sampling strategy

All records of adolescent patients who were admitted between January 2015 and December 2019 were examined. Cases were defined as patients who had been admitted to Weskoppies Hospital adolescent unit during the 5-year study period and who were readmitted at any point during the defined time period. Controls included patients admitted to the same unit who were not readmitted during the follow-up period. Researchers excluded non-adolescent patients, in accordance with the International Association for Child and Adolescent Psychiatry and Allied Professions’ definition of adolescence,^23^ that is, those who were younger than 11 years and older than 18 years. Further exclusions included patients with missing files, and patients who were ineligible for readmission, such as in the case of death. If a patient was admitted before 2015, this was noted as their age of first admission; however, the rest of the data collected were limited to the 5-year period being studied.

### Data collection

Fixed demographic details were recorded, including gender, age at present admission, primary *Diagnostic and Statistical Manual of Mental Disorders, Fifth Edition* (DSM-5) diagnosis, comorbidities, and level of education (mainstream or special schooling).

The primary DSM-5 diagnoses included neurocognitive disorders, schizophrenia spectrum disorders, bipolar and related disorders, major depressive disorder and other disorders. The exposure variables included non-adherence to treatment plan, as evidenced by follow-up to outpatient appointments (doctor’s appointments), family and patient reporting or, where available, serum drug levels; substance use, as reported by the patient or their family members or evidenced in urine drugs of abuse screen where possible; suicide attempt or ideation; parental mental illness; home environment factors; interpersonal relationship difficulties; recent personal loss; and childhood sexual assault history. After collection, the data were anonymised and captured electronically on an Excel spreadsheet for statistical analysis.

A sample size calculation was conducted using a Pearson’s chi-squared test which aimed to detect a 20% difference in the distribution of a dichotomous modifiable risk factor between the two strata with equal variance. Based on expert opinion, we assumed a non-readmission group to readmission group ratio of 3:1. Setting the significance level at 5% and power of 80%, researchers calculated that 176 participants were needed for the study.

### Data analysis

An exploratory analysis was done to examine the distribution and pattern of variables between cases and controls. Bivariate analysis was done using hypothesis tests (unpaired *t*-test for normally distributed continuous variables and chi-squared tests for categorical variables) to investigate differences between predictor variables’ distributions in the admission and readmission groups. Thereafter, the additive effect of risk factors was explored simultaneously using a generalised linear regression model. Researchers first built a saturated model producing adjusted odds ratios (aOR) and reduced the number of predictors included based on goodness-of-fit parameters. Univariate logistic regression produced unadjusted odds ratios and accompanying 95% confidence intervals (CI). Finally, time to readmission and produced non-parametric survival analysis producing Kaplan–Meier survival functions was considered to further examine the data.

### Ethical considerations

Permission for access to the clinical notes was granted by the Chief Executive Officer of Weskoppies Psychiatric Hospital before the commencement of data collection. This was a case-control study involving a retrospective review of patient clinical files; thus, no personal patient consent was required. Each file was allocated a study number to anonymise data. The Faculty of Health Sciences Research Ethics Committee, University of Pretoria, approved the study (Reference number: 522/2021). The study was also submitted to The National Health Research Database of South Africa for approval (Reference number: GP 202205 079).

## Results

### Demographics

During the study period, 516 patients were admitted to the child and adolescent unit. Of these, 164 patients were younger than 11 years, 6 files were not traceable in records and 1 patient demised shortly after first admission; therefore, these files were excluded. Thus, 345 adolescent patients were included in this study (*n* = 345). Of these, 247 patients were not readmitted, while 98 were readmitted within the 5-year follow-up period. There were 143 (41%) female patients, and the mean age at first admission was 14.03 years. The primary diagnosis of patients was equally distributed across the readmitted and non-readmitted groups, with the exception of patients with bipolar and related disorder, of which a greater proportion were readmitted (33.7%) compared to patients who were not readmitted (24.3%). There was a smaller proportion of patients with major depressive disorder in the readmitted group (14.3%) compared to the non-readmitted group (23.5%). More than half of all patients had multiple psychiatric diagnoses (*n* = 206, 60%). In all, 77 patients attended special schooling (*n* = 77, 22%), while 268 patients attended mainstream schooling (*n* = 268, 78%). The demographic factors are summarised in Table 1.

**TABLE 1:** Characteristics of adolescent admissions at Weskoppies Hospital between 2015 and 2019 (*N* = 345).

Characteristic	*n*	%
**Sex of patient**		
Male	202	59
Female	143	41
**Age at first admission**	14.03	2.47
**Comorbidity**		
Not present	139	40
Present	206	60
**Educational level**		
Special schooling	77	22
Mainstream schooling	268	78
**Documented history of non-adherence**		
Adherent	274	82
Non-adherent	62	18
Unknown	9	-
**Substance abuse history**		
No evidence of substance use	212	62
Occasional or recreational substance use	63	18
Meets DSM-5 criteria for a substance use disorder	67	20
Unknown	3	-
**History of suicidal ideation**		
No evidence of suicidal ideation or attempt	164	48
Suicidal ideation	37	11
Self-harm	40	12
Suicidal ideation and attempt	102	30
Unknown	2	-
**Parental mental illness**		
None	183	58
One or both parents	131	42
Unknown	31	-
**Parent–child conflict at home**		
Stable home environment	106	36
Verbal and/or emotional and/or physical conflict	38	13
Child institutionalised (orphanage or foster care)	63	22
Conflicts at home because of child’s disruptive behaviour	84	29
Unknown	54	-
**Interpersonal relationship difficulties**		
No interpersonal relationship difficulties	106	31
Conflict reported	238	69
Unknown	1	-
**Personal loss**			113	33
Unknown	1	-
**Documented childhood sexual assault**			65	19
Unknown	3	-

DSM-5, Diagnostic and Statistical Manual of Mental Disorders, Fifth Edition.

**FIGURE 1:**
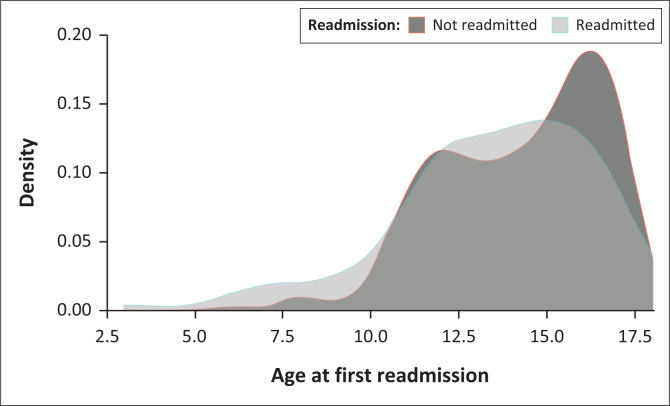
Age of first admission (years) in the readmitted versus not readmitted group. The patients included adolescent psychiatric patients admitted between 2015 and 2019 at Weskoppies Hospital in South Africa.

Adolescents in the readmitted groups were significantly younger at first admission compared to adolescents in the non-readmitted group (13.46 vs 14.26, *p* = 0.016; Figure 1). A univariate analysis revealed that the odds of being readmitted decreased by 0.88 per year of increasing age at first admission (95% CI = 0.80–0.97, *p* = 0.00741) (see Table 2).

**TABLE 2:** Bivariate analysis of risk factors for readmission of adolescent psychiatric inpatients at Weskoppies Hospital, South Africa, between 2015 and 2019.

Characteristics	Overall (*N* = 345)				Not readmitted (*N* = 247)				Readmitted (*N* = 98)				*P*
	*n*	%	Mean	s.d.	*n*	%	Mean	s.d.	*n*	%	Mean	s.d.	
Gender = female (%)	143.0	41.4	-	-	100.0	40.5	-	-	43.0	43.9	-	-	0.58
Current age	-	-	14.57	2.04	-	-	14.49	2.08	-	-	14.74	1.94	0.30
First admission age	-	-	14.03	2.47	-	-	14.26	2.28	-	-	13.46	2.81	0.02
Primary diagnosis (%)	-	-	-	-	-	-	-	-	-	-	-	-	0.23
Learning difficulty or disability and behavioural problems	67.0	19.4	-	-	48.0	19.4	-	-	19.0	19.4	-	-	-
Schizophrenia spectrum	62.0	18.0	-	-	46.0	18.6	-	-	16.0	16.3	-	-	-
Bipolar disorder and/or BD and/or BMD	93.0	27.0	-	-	60.0	24.3	-	-	33.0	33.7	-	-	-
Major depressive disorder or MDD	72.0	20.9	-	-	58.0	23.5	-	-	14.0	14.3	-	-	-
Other	51.0	14.8	-	-	35.0	14.2	-	-	16.0	16.3	-	-	-
Comorbidity present (%)	206.0	59.7	-	-	142.0	57.5	-	-	64.0	65.3	-	-	0.20
Education: Mainstream schooling (%)	268.0	77.7	-	-	197.0	79.8	-	-	71.0	72.4	-	-	0.14
Non-adherence: Yes (%)	62.0	18.5	-	-	25.0	10.5	-	-	37.0	38.1	-	-	< 0.001
Substance use (%)	-	-	-	-	-	-	-	-	-	-	-	-	0.87
No evidence of substance use	212.0	62.0	-	-	154	62.9	-	-	58.0	59.8	-	-	-
Occasional or recreational substance use	63.0	18.4	-	-	44.0	18.0	-	-	19.0	19.6	-	-	-
Meets criteria for a substance use disorder	67.0	19.6	-	-	47.0	19.2	-	-	20.0	20.6	-	-	-
Suicidal ideation (%)	-	-	-	-	-	-	-	-	-	-	-	-	0.36
No evidence of suicidal ideation or attempt	164.0	47.8	-	-	123.0	50.2	-	-	41.0	41.8	-	-	-
History of suicidal ideation	37.0	10.8	-	-	27.0	11.0	-	-	10.0	10.2	-	-	-
Self-harm	40.0	11.7	-	-	29.0	11.8	-	-	11.0	11.2	-	-	-
History of suicide ideation and attempt	102.0	29.7	-	-	66.0	26.9	-	-	36.0	36.7	-	-	-
Parental mental illness: One or more parent mental illness reported (%)	131	41.7	-	-	84.0	37.5	-	-	47.0	52.2	-	-	0.015
Home environment par child conflict (%)	-	-	-	-	-	-	-	-	-	-	-	-	0.824
Stable home environment	159.0	46.2	-	-	116.0	47.2	-	-	43.0	43.9	-	-	-
Verbal and/or emotional and/or physical conflict	38.0	11.0	-	-	25.0	10.2	-	-	13.0	13.3	-	-	-
Adolescent institutionalised (orphanage or foster care)	63.0	18.3	-	-	44.0	17.9	-	-	19.0	19.4	-	-	-
Conflicts at home because of adolescent’s disruptive behaviour	84.0	24.4	-	-	61.0	24.8	-	-	23.0	23.5	-	-	-
Interpersonal relationship difficulties: conflict reported	185.0	53.8	-	-	130.0	52.8	-	-	55.0	56.1	-	-	0.60
Personal loss: Yes (%)	113.0	32.8	-	-	78.0	31.7	-	-	35.0	35.7	-	-	0.47
Childhood sexual assault: Yes (%)	65.0	19.0	-	-	41.0	16.7	-	-	24.0	24.7	-	-	0.088

When comparing the other demographic factors, a univariate analysis revealed that the odds of readmission were higher for adolescents who attended special schooling versus those in mainstream schooling (odds ratio [OR]: 1.61; 95% CI = 0.93–2.77, *p* = 0.0887).

### Exposure variables

**FIGURE 2:**
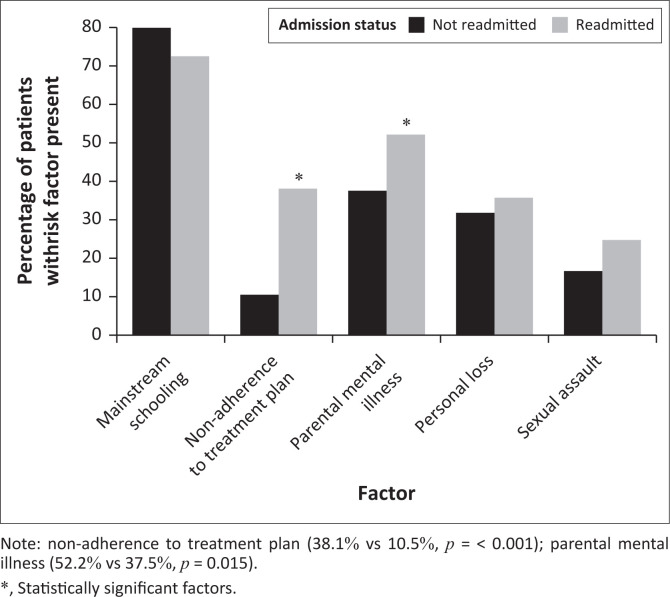
Factors affecting readmission of adolescent psychiatric inpatients at Weskoppies Hospital in South Africa between 2015 and 2019.

In total, 274 patients reportedly adhered to their treatment plans post-discharge (82%), whereas 62 (18%) were non-adherent. Researchers were unable to comment on nine of the patients’ adherence because of incomplete reporting in the patient records. Comparatively to the non-readmitted group, bivariate analysis showed that patients in the readmitted group had a much higher rate of non-adherence to treatment (38.1% vs 10.5%, *p* = < 0.001; Figure 2). Despite adjusting for other variables, the odds of readmission were 9.29 times higher if a patient was non-adherent (95% CI = 4.31–19.99, *p* = < 0.001; Table 3).

**TABLE 3:** Unadjusted and covariate-adjusted risk factors for readmission adolescent psychiatric inpatients at Weskoppies Hospital, South Africa, between 2015 and 2019.

Variable	Unadjusted (univariate analysis)			Adjusted (full model)		
	Odds ratio (OR)	95% CI	*P*	Adjusted odds ratio (aOR)	95% CI	*P*
**Gender**						
Male (reference)	1	-	-	1	-	-
Female	1.15	0.72–1.84	0.564	1.19	0.61–2.31	0.61
**Age at first admission**	0.88	0.80–0.97	0.007	0.83	0.72–96	0.108
**Primary diagnosis**						
Neurocognitive disorder (reference)	1	-	-	1	-	-
Schizophrenia spectrum	0.88	0.4–1.91	0.74	1.09	0.35–3.35	0.88
Bipolar and related disorder	1.39	0.70–2.74	0.34	1.33	0.49–3.56	0.58
Major depressive disorder	0.61	0.28–1.34	0.22	0.41	0.13–1.30	0.13
Other	1.15	0.52–2.56	0.72	1.38	0.48–3.98	0.55
**Presence of comorbidity**						
Not present (reference)	1	-	-	1	-	-
Present	1.39	0.86–2.26	0.183	1.17	0.60–2.27	0.64
**Education**						
Mainstream schooling	1	-	-	1	-	-
Special schooling	1.61	0.93–2.77	0.09	1.30	0.57–2.97	0.53
**Home environment and parent–child conflict**						
Stable home environment	1	-	-	1	-	-
Verbal and/or emotional and/or physical conflict reported	1.4	0.66–2.99	0.38	1.70	0.68–4.24	0.25
Adolescent institutionalised	1.16	0.61–2.21	0.64	0.97	0.39–2.41	0.95
Conflicts at home because of adolescent’s disruptive behaviour	1.02	0.56–1.84	0.96	0.65	0.31–1.41	0.28
**Documented non-adherence**						
Adherent (reference)	1	-	-	1	-	-
Non-adherent	5.28	2.95–9.45	< 0.001	9.29	4.31–19.99	< 0.001
**Substance use**						
No evidence of substance use (reference)	1	-	-	1.00	-	-
Occasional and/or recreational substance use	0.89	0.48–1.62	0.69	1.29	0.57–2.89	0.54
Meets criteria for a substance use disorder	1.01	0.48–2.15	0.97	1.14	0.48–2.72	0.76
**Suicidal history**						
No evidence of suicidal ideation and/or attempt (reference)	1	-	-	1	-	-
History of suicidal ideation	1.11	0.50–2.49	0.80	1.30	0.48–3.51	0.6
Self-harm	1.13	0.52–2.48	0.75	1.04	0.37–2.92	0.94
History of suicide ideation and attempt	1.64	0.96–2.80	0.073	1.81	0.84–3.90	0.13
**Parental mental illness**						
No history of parental mental illness (reference)	1	-	-	1	-	-
One or both parents mental illness reported	1.82	1.11–2.99	0.017	1.87	1.02–3.41	0.04
**History of personal loss**						
None reported (reference)	1	-	-	1.00	-	-
Personal loss reported	1.2	0.73–1.96	0.48	1.72	0.91–3.26	0.08
**History of childhood sexual assault**						
None reported (reference)	1	-	-	1	-	-
History of sexual assault	1.64	0.92–2.89	0.09	0.95	0.42–2.41	0.91

There was a similar proportion of patients who used substances, either recreationally or diagnosed with a substance use disorder in the readmitted and non-readmitted groups (40.6% vs 37.2%). There was a larger proportion of patients in the readmitted group who had a history of suicide attempt (36.7% vs 26.9%), but the difference was not statistically significant.

A significantly substantial proportion of readmitted patients had one or both parent(s) who suffered from a mental illness (52.2% vs 37.5%, *p* = 0.015). And in turn, patients whose parent(s) suffered from mental illness had a higher risk for readmission (OR = 1.87; 95% CI = 1.02–3.41, *p* = 0.04).

A large proportion of patients had a history of parent–child conflict (42%) or had been removed from their home and institutionalised (22%). Most patients also reported interpersonal relationship difficulties (69%), and many had experienced the loss of a loved one (33%); however, these factors did not differ significantly between the readmitted and non-readmitted groups. Finally, 65 patients (*n* = 65, 16%) had a documented history of childhood sexual assault. A larger proportion of said adolescents had been readmitted (24.7% vs 16.7%, *p* = 0.088). It was demonstrated that a history of sexual assault increased the risk of readmission by 1.64 (95% CI = 0.92–2.89, *p* = 0.0908).

Kaplan–Meier survival analyses revealed that none of the investigated factors significantly reduced the length of time between successive admissions. The relationship between length of admission and risk for readmission was examined; however, longer period of admission was not found to significantly affect the risk for readmission.

## Discussion

In this case-control study, we investigated the factors that are associated with the readmission of adolescents to a specialist adolescent psychiatric unit at Weskoppies Hospital, with the aim of identifying risk factors for readmission, particularly those that may be modifiable. The most prominent risk factors were age at first admission, adherence to a post-treatment plan and having parent(s) with a history of mental illness.

The most significant finding of our study was the association between non-adherence and the risk for readmission, with the odds of readmission increasing by 9.29 when patients did not adhere to the treatment plan. This is an already well-established risk factor discussed in several studies.^8^ Our findings reiterate the importance of regularly updating treatment guidelines to include appropriate post-discharge follow-up and educating patients on the importance of adherence. In preparing post-discharge plans, it is imperative to consider that multiple factors may influence medication non-adherence such as severity of illness, comorbid substance abuse, adverse side effects of medication and inadequate social support, including unavailability of family to support the appropriate use of medication.^24^ This information should be used to personalise treatment plans accordingly. Improving access to robust community mental health facilities for rehabilitation and further support of patients would also increase treatment adherence. This would ensure regular follow-up and pre-emptive management of complications and referral to specialist services if needed. These facilities are also crucial for psychiatric and medical screening in adolescents.^25^ Community mental health services should ideally offer parent training programmes in order to enhance parent skills to improve the holistic well-being of the family unit. Assertive community treatment among adolescents is a promising evidence-based approach to improve outcomes in those with severe mental illness. The benefits of which include reducing the duration and frequency of hospital admissions.^26^

The study found that the younger age of first admission increased the risk for readmission, which was in accordance with similar studies conducted.^7,8^ It was hypothesised that this could be because of earlier age of illness onset being associated with an increased likelihood of severe illness. Alternatively, the view among healthcare providers that mental illnesses typically develop in older adolescence to early adulthood may result in underdiagnosis in children and therefore longer periods of untreated illness and greater illness severity.

Studies have shown that intervention during the early stages of a disorder may help reduce illness severity as well as prevent secondary disorders.^27^ This highlights the benefit of early screening and intervention for children and adolescents to avoid the need for initial admission.

In this study, male and female patients had similar rates of readmission. Attending special schooling increased the odds of readmission by 1.61. This could be related to adolescents with more severe symptoms requiring special schooling or being excluded from mainstream schooling because of higher rates of behavioural disturbance.^28^

Clinically, the patients’ primary diagnoses were not associated with being readmitted. Interestingly, a previous suicide attempt seemed to increase the risk for readmission, but not by a significant margin. This finding is in contrast with other studies that found suicidality to be a main predictor for readmission.^9,13,15^ The authors infer that the reason for this was that 53% of all the patients in our study had some history of suicidal ideation, self-harm or suicide attempt, and therefore, numbers were high in both the single admission and readmitted groups.

The above inference is supported by findings of the Youth Risk Behaviour Surveillance System which was used to collect data from South African schools in 2002, 2008 and 2013. The results of this survey found that 17% of South African learners had made one or more suicide attempts in their lifetime.^29^ This number is significantly lower than the overall 30% of adolescents in our study who had attempted suicide. This finding suggests that adolescents with mental illness have increased susceptibility to suicidal behaviour and highlights the importance of screening for suicidal ideation at each patient interaction.

A history of sexual assault was found to increase the risk of readmission; however, this finding was not statistically significant. The authors propose that this may be an important area of future study as the number of patients who experienced sexual assault may well have been under-reported when considering that those with mental illness are statistically more vulnerable than the general population and may be less likely to report the assault.^30^ This is an important risk factor to target as sexual assault is strongly linked to mental illness. The Optimus study conducted in South Africa over the same time as our research found that adolescents who reported having been sexually assaulted were more than twice as likely to report anxiety and depression, and three times as likely to report post-traumatic stress disorder (PTSD) symptoms, as other young South Africans.^31^ A prior history of sexual assault has been associated with future chronic medical conditions as well.^32^

This study corroborated previous findings that adolescents with a family history of mental illness have a higher risk of being readmitted.^17,18^ While this is a non-modifiable risk factor, it should be considered during the screening process and may assist in the early identification and management of mental illness.

Other environmental factors, including interpersonal relationship problems, parent–child conflict and a history of personal loss, did not appear to significantly influence the risk for readmission. However, most of the patient population had experienced some degree of home conflict and 53.8% reported interpersonal relationship difficulties. This is important when considering that interpersonal relationships influence adolescent development and how stressful relationships may result in general maladjustment and feelings of loss or rejection.^33^

Interpersonal relationship difficulties may increase the risk of developing mental illness, even if they do not significantly influence the need for multiple admissions.

Strengths of this study include a large study population with 5 years of admissions. The use of univariate and aOR resulted in examining marginal and adjusted effects and producing more robust findings. Finally, our results add further insight into readmission in the South African setting where there is a paucity of research on this topic. The study is limited, albeit minimally, by incomplete data. Firstly, the researchers did not exclude any participants from the analysis because of missing data as the level of missingness was minor. The missingness was assumed to be missing completely at random when encountered, and pairwise deletion was used in the analysis. Secondly, the follow-up period was limited to 5 years, and we did not consider readmissions that may have occurred after the study period. Moreover, while we did include readmissions to other psychiatric hospitals if patients reported them during follow-up, those who did not report may have been recorded as non-readmitted patients. Thirdly, a self-developed scale for recording data was used, which may hinder the reproducibility of the study. Fourthly, only patients who were admitted to a specialised adolescent psychiatric unit was included and did not include any patients from primary healthcare facilities or general hospital settings, which may limit the generalisability of results. Finally, non-readmission was used as a measure of stability; however, this may not necessarily equate to full symptomatic and functional recovery.

## Conclusion

The findings from our study suggest that the readmission of adolescent psychiatric patients is associated with numerous factors. The factors associated with readmission in South African adolescents are similar to those reported internationally, and this study contributes to the growing literature on this topic.

Healthcare professionals should consider the impact of family history on the development of mental illness, irrespective of the diagnosis. Findings also highlight the impact of admission at a younger age on future risk of readmission. Future research could investigate the correlation between family history and early age of onset of illness.

Lastly, the study emphasises the importance of adherence to treatment plans post-discharge. Strengthening of community mental health services could potentially aid in screening for psychopathology in youth and reducing the duration and frequency of hospital admissions.

The World Health Organization (WHO) emphasises that failing to address factors that affect adolescent mental health results in concerns extending to adulthood and limiting opportunities for patients to lead fulfilling lives.^34^ The authors vehemently concur and advocate that further research in this field would be of great benefit in achieving longstanding improved patient outcomes.
